# *Limosilactobacillus reuteri* metabolites modulate immune pathways and intestinal barrier repair after 5 fluorouracil exposure

**DOI:** 10.1038/s41598-026-45524-y

**Published:** 2026-04-02

**Authors:** Gintare Lasaviciute, Marta López Plana, Sofia Sundberg Örtegren, Sevasteia Telli, Symeon Kourmoulakis, Ludwig Ermann Lundberg, Kenny Lidberg, Oshadi Peiris, Indranil Sinha, Ann-Beth Jonsson, Stefan Roos, Anna Nilsson, Manuel Mata Forsberg, Eva Sverremark-Ekström

**Affiliations:** 1 Department of Molecular Biosciences, The Wenner-Gren Institute, Stockholm University, Stockholm, 106 91 Sweden; 2 Department of Molecular Sciences, Uppsala BioCenter, Swedish University of Agricultural Sciences, Uppsala, Sweden; 3 BioGaia, Stockholm, Sweden; 4 Department of Women’s and Children’s Health, Pediatric Oncology Unit, Karolinska Institute, Stockholm, Sweden

**Keywords:** Chemotherapy, Probiotics, *Limosilactobacillus reuteri*, Exopolysaccharides, Extracellular membrane vesicles, Epithelial cells integrity, Cancer, Cell biology, Immunology, Microbiology

## Abstract

Antimetabolites such as 5 fluorouracil are known to induce inflammation in the gut and oral cavity, underscoring the need for strategies that mitigate chemotherapy-associated toxicity. The aim of this study was to determine whether secreted components from the probiotic bacterium *Limosilactobacillus reuteri* DSM 17938, specifically cell-free supernatant, exopolysaccharides, and extracellular membrane vesicles, can support epithelial barrier recovery following 5 fluorouracil-induced injury. Exposure to 5 fluorouracil impaired viability, metabolic activity, and barrier integrity, and shifted the functional responses of Caco-2 cells toward increased inflammation. Stimulation with exopolysaccharides after removal of 5 fluorouracil significantly improved barrier integrity in both enterocyte-like Caco-2 cells and primary human intestinal epithelial cells, while paradoxically inducing an inflammatory protein profile in the enterocyte-like cells. Transcriptomic analysis revealed that exopolysaccharides modulate gene programs associated with extracellular matrix organization and structural remodelling. Furthermore, cell-free supernatant, membrane vesicles, and exopolysaccharides differentially influenced monocyte polarization pathways when monocytes were cultured with supernatant from 5 fluorouracil-exposed Caco-2 cells. Together, these findings demonstrate that bacterial metabolites such as exopolysaccharides influence intestinal barrier recovery upon inflammation and activate immune cell recruitment that could have consequences for the intestinal epithelial integrity during inflammation.

**Supplementary Information:**

The online version contains supplementary material available at 10.1038/s41598-026-45524-y.

## Introduction

Although advanced chemotherapy regimens have improved survival rates worldwide, undesirable side effects are imminent in cancer patients. Chemotherapeutic agents directly impair epithelial barrier integrity leading to the release of inflammatory mediators, bacterial translocation, dysbiosis and gastrointestinal dysfunction^1,2^. 5 Fluorouracil (5 FU) is routinely used against a wide range of malignancies including breast, stomach, and colon cancers. It functions as an antimetabolite and it is known to induce mucositis, an inflammation of the mucous membranes lining the gut and oral cavity^3,4^. Adverse effects of 5 FU administration restrict the anticancer treatment efficiency, as toxicity management options are limited.

Increasing evidence suggests that selective gut probiotics might be capable of alleviating chemotherapy-induced toxicity, especially highlighting bifidobacteria and lactobacilli bacteria^5,6^. Lactobacilli are Gram-positive, facultative anaerobes frequently used as probiotics. For decades, lactobacilli comprised a rather diverse group of bacteria, while only recently the *Lactobacillus* genus was subdivided into 25 genera based on the genomic and physiological characteristics^7^. Beneficial effects of lactobacilli administration during anticancer treatment have been emphasized in several studies. Mice subjected to intraperitoneal injections with 5 FU and simultaneously fed a combination of two *Limosilactobacillus reuteri* strains, previously known as *Lactobacillus reuteri*, DSM 17938 (from now on referred to as LR) and ATCC PTA 5289, show reduced oxidative stress via nuclear factor E2-related factor 2 (Nrf-2) and dampened inflammation in the oral mucosa^8^. *Lactobacillus delbrueckii subsp. lactis* in combination with *Bifidobacterium longum* and *Enterococcus faecium* have been shown to reduce the severity of radiotherapy-induced oral mucositis in cancer patients^9^, while *Lacticaseibacillus rhamnosus* GG (LGG), former *Lactobacillus rhamnosus* GG, protected the gut epithelium from radiotherapy-induced cytotoxicity in mice^10^. Nevertheless, most studies have previously focused on live or heat-killed bacteria lacking comprehensive investigation on soluble metabolites, which might possess immunomodulatory effects as they penetrate the intestinal barrier^11,12^. Further, the administration of soluble bacterial components rather than live microorganisms after chemotherapy might be a safer option for immunocompromised cancer patients.

Here, we studied whether LR-derived cell-free supernatant (CFS), exopolysaccharides (EPS), and extracellular membrane vesicles (MV) can advance the repair of 5 FU-induced damage in intestinal epithelial cells (IEC). We also examined how the different microbial exposures altered the inflammatory response of intestinal epithelial cells, and whether these chemotherapy- and microbe-exposed cells subsequently affected macrophage differentiation^13^. We show that 5 FU exposure significantly affected cellular viability, metabolism, barrier integrity and functional responses. The addition of EPS significantly improved epithelial barrier integrity, although pro-inflammatory proteins were significantly upregulated. The same effects were observed using enterocyte-like Caco-2 cells and primary human small intestinal epithelial cells (hSIEC). An analysis of the transcriptome of treated Caco-2 cells, showed that EPS stimulation modified genes associated with the extracellular matrix organization and retinoic acid (RA) biosynthesis, both of which are important for intestinal barrier maintenance. When examining if the addition of probiotic bacteria components after chemotherapy also impacts immune cells, which are normally in close contact with IEC, we observed that EPS promoted M1-like macrophages, indicating an immune responsive cell phenotype. In contrast, the supernatant from 5 FU exposed and CFS stimulated Caco-2 cells polarized monocytes into M2-like macrophages, while MV indirectly polarized cells with a mixed phenotype of both, M1-like and M2-like, macrophages. Collectively, our results indicate that EPS modulate epithelial responses to chemotherapy-induced stress in ways that could be relevant for mitigating mucosal injury.

## Materials and methods

### Ethics statement

All methods were carried out in accordance with relevant guidelines and regulations. Intestinal epithelial cell experiments were performed exclusively with commercially available cell line or primary intestinal cells, as described below. Peripheral blood mononuclear cells for monocyte isolation were obtained as residual material from the preparation of red blood cells and platelets from whole-blood donations by anonymous, healthy donors. Because the biological material cannot be traced back to any individual, the Swedish Ethical Review Authority has determined that the project does not fall under the Ethical Review Act, and no ethical permit is required.

### Cell lines and their culture conditions

Authenticated human colon adenocarcinoma cell line Caco-2 (RRID: CVCL_0025, ATCC catalogue no. HTB-37) was purchased from Sigma-Aldrich. The cell line was tested for mycoplasma contamination using MycoAlert Mycoplasma Detection kit and MycoAlert Assay Control set (both from Lonza), and it was confirmed to be free of contamination throughout the experiments. Caco-2 cells were cultured in Minimum Essential Medium Eagle (EMEM) supplemented with 10% FBS (both from Sigma-Aldrich), 2 mM L-glutamine, 100 U/ml penicillin and 100 µg/ml streptomycin (all from Cytiva). For Caco-2 cell differentiation to enterocyte-like cells, Dulbecco’s Modified Eagle medium (DMEM) with high glucose (Thermo Fisher Scientific) was supplemented with 1.15 µg/ml RA (Sigma Aldrich), 10% FBS, 2 mM L-glutamine, 100 U/ml penicillin and 100 µg/ml streptomycin (complete DMEM medium). Of note, RA was present in the culture medium throughout all experimental phases, including 5 FU exposure and subsequent EPS treatment, to maintain in vivo-like conditions. Cells were incubated at 37 °C with 5% CO_2_ throughout the experiments.

### Generation of LR-CFS

*Limosilactobacillus reuteri* DSM 17938 (LR) was a kind gift from BioGaia AB (Stockholm Sweden). The bacteria were grown on Rogosa agar plates for 24 h whereby a single colony was inoculated into De Man, Rogosa and Sharpe (MRS) medium and grown as still cultures overnight. The bacteria were centrifugated, resuspended in RPMI-1640 medium supplemented with 18 g/l glucose and 20% FBS at OD_600nm_ of 0.2 and were grown as still cultures for 48 h at 37 °C, 5% CO_2_. Following another centrifugation, the pH was neutralized with 5 M NaOH. The CFS was collected, 0.2 μm filtered and stored at -20 °C.

### Generation of LR-derived EPS and MV

For the MV preparation, LR DSM 17938 was grown in MRS broth for 24 h at 37 °C. To separate bacterial cells from the supernatant, they were centrifugated at 5000x*g* and 4 °C for 10 min, followed by the second centrifugation at 1000x*g* and 4 °C for 10 min. The supernatant was then collected, filtered using 0.45 μm pore filter (Millipore), and concentrated using Amicon Ultra filter unit with a MwCO of 100 kDa. The supernatant was then loaded on top of 12% sucrose cushion with 50 mM Tris buffer at pH 7.2 and with the volume ratio of 5:1. Next, the supernatant was centrifugated using Optima L-80 XP ultracentrifuge (Beckman coulter) at 118,000x*g* and 4 °C for 3 h. The supernatant was then removed, and the pellet was resuspended in PBS and centrifugated again at 118,000x*g* and 4 °C for 3 h. Pelleted MVs were then aliquoted and stored at -70 °C. The concentration of MV was quantified by nanoparticle tracking analysis (NTA) using the NanoSight NS300 system. With a 488 nm laser and a high sensitivity sCMOS camera, videos were collected and analysed using the NTA software v.3.4 to generate a size distribution curve and a concentration was calculated.

EPS from LR were prepared as follows; LR DSM 17938 were grown in MRS broth for 16 h at 37 °C without agitation. The bacteria were then inoculated in the *Lactobacillus* carrying medium containing 2% of sucrose and were grown for 24 h at 37 °C without agitation. To separate bacterial cells from the supernatant, the cultures were centrifugated at 4000x*g* for 10 min, the pellet was discarded and proteins in the supernatant were precipitated using trichloroacetic acid at 20% final concentration. Following incubation for 15 min at 2 °C, the supernatant was centrifugated at 25,000x*g* for 30 min at 4 °C. The pellet was discarded and acetone, equivalent to one volume of the supernatant, was added. Following incubation at 2 °C for 24 h, the suspension was centrifugated at 25,000x*g* for 30 min at 4 °C. Pelleted EPS were resuspended in water and dried in a Coolsafe freezer dryer (Scanvac), after which, EPS were stored at -20 °C. The obtained weight of EPS after freeze-drying was 110 mg. To measure carbohydrate concentration using phenol sulphuric acid, 5 mg of purified EPS was dissolved in 5 ml dH_2_0 after which it was diluted 10 times and to a total volume of 1 ml. Glucose standards at a concentration of 0.01 mg/ml, 0.025 mg/ml, 0.05 mg/ml, 0.075 mg/ml and 0.1 mg/ml was used. 500 µl of 5% phenol solution and 2.5 ml of 96% sulphuric acid were added followed by vortex at slow speed. The samples were then incubated at 20 °C for 30 min. Absorbance was measured at 490 nm and the EPS concentrations were calculated using a standard curve that was obtained from the glucose standards. The procedure was performed in duplicates. The total carbohydrate concentration in the purified EPS sample was 90%. A partial characterization of the purified EPS has been published previously^14^.

### Undifferentiated Caco-2 cell culture with the CFS from LR

A total of 0.3 × 10^6^ Caco-2 cells/well were seeded in 12-well plates and were left to incubate for 24 h. Then cells were stimulated with 10% of CFS from LR or were left unstimulated, (prior-chemotherapy conditions). After 24 h, the medium was removed and cells were exposed to 50 µg/ml of 5 FU or 1 µg/ml of Doxorubicin (Doxo) (both from Sigma Aldrich) or were left untreated as a control. Following 24 h incubation, cells were washed twice with PBS and then were resuspended in fresh medium with or without CFS from LR (post-chemotherapy conditions). Cells were rested for additional 24 h and then were collected for downstream applications.

### MTT assay

CyQUANT MTT assay (Invitrogen) was used according to the manufacturer’s instructions. Briefly, 25 × 10^3^ Caco-2 cells/well were seeded in 96-well plates. Following 3 days of incubation, cells were washed twice with PBS and chemotherapy drugs were added as following: 0,5 µg/ml, 1 µg/ml or 2 µg/ml of Doxo, and 20 µg/ml, 50 µg/ml or 100 µg/ml of 5 FU, in triplicates. Cells without chemotherapy exposure served as the control. Following 24 h of incubation, the medium was removed and 110 µl/well of fresh medium containing 10 ml of 12-mM MTT stock solution was added. Following 2.5 h of incubation at 37 °C, medium was removed and formazan crystals were dissolved using 100 µl of DMSO (Sigma Aldrich). The absorbance was measured at 540 nm using SpectraMax i3x plate reader (Molecular Devices Corp.). The viability of cells was expressed as the percentage relative to the untreated cells.

### Fluorescent microscopy

Caco-2 cells were seeded at 0.7 × 10^6^ cells/well in 12-well collagen-coated poly-D-lysine glass-bottom plates (MatTek) and were left to incubate for a day until confluence reached approximately 90%. Then the cells were exposed to 50 µg/ml of 5 FU or 1 µg/ml of Doxo for 24 h, while non-exposed cells served as the control. Cells were then washed with PBS twice and were left to rest for additional 24 h. The staining of tight junction proteins (TJP) was performed as following; cells were fixed with 4% paraformaldehyde (Sigma Aldrich) for 15 min, permeabilized with 0.25% Triton X-100 for 10 min followed by blocking with 5% BSA for 1 h. Occludin (OCLN) monoclonal antibody (Invitrogen, clone OC-3F10) and zonula occludens-1 (ZO-1) monoclonal antibody (Invitrogen, clone ZO1-1A12) were diluted in 1% BSA at 1 µg/ml final concentration and were added to stain TJP. The DNA damage marker γH2AX (Cell Signaling Technology, clone 20E3) was diluted 1:400 in 1% BSA and was added at the same time. Following incubation overnight at 4 °C, goat anti-mouse IgG (H + L) highly cross-absorbed secondary antibody labelled with Alexa Fluor™ plus 488 (Invitrogen, #A32723) was diluted in 1% BSA at 1 µg/ml and goat anti-rabbit IgG (H + L) highly cross-absorbed secondary antibody labelled with Alexa Fluor™ 647 (Invitrogen, #A21244) was diluted in 1% BSA at 4 µg/ml. Cells were incubated with secondary antibodies for 45 min at room temperature. Nuclei were stained with DAPI (Invitrogen, #62248) at 1 µg/ml in 1% BSA for 10 min. The images were taken with 20x or 40x magnification using a ZEISS LSM 800 Airy scan confocal microscope. The analysis was made using ZEN blue 2.3 software.

### Flow cytometry

Caco-2 cells were seeded at 0.7 × 10^6^ cells/well in 12-well plates and were left to incubate for a day until confluence reached approximately 90%. Cells were then exposed to 50 µg/ml of 5 FU or 1 µg/ml of Doxo for 24 h, while non-exposed cells served as the control. Following 24 h incubation, cells were washed twice with PBS and were left to rest for a day. TrypLE™ express solution (Gibco™) was used to detach cells. The fixable viability stain 780 (BD Biosciences) was added for 15 min at 4 °C to identify non-viable cells. Next, cells were fixed with 4% paraformaldehyde for 15 min at room temperature followed by permeabilization with 0.25% Triton X-100 for 10 min. To block non-specific antibody binding, cells were incubated with 5% BSA for 45 min. Cells were stained with OCLN monoclonal antibody (Invitrogen, clone OC-3F10), claudin-1 (CLDN1) monoclonal antibody (Invitrogen, clone 2H10D10), and ZO-1 monoclonal antibody (Invitrogen, clone ZO1-1A12), all diluted in flow cytometry buffer (containing PBS, 2 mM EDTA and 0.1% BSA) at 1 µg/ml final concentration, for 1 h, at 4 °C. The DNA damage marker γH2AX (Cell Signaling Technology, clone 20E3) was diluted 1:400 and was added at the same time. Goat anti-mouse IgG (H + L) highly cross-absorbed secondary antibody labelled with Alexa Fluor™ plus 488 (Invitrogen, #A32723) was diluted in flow cytometry buffer at 1 µg/ml and goat anti-rabbit IgG (H + L) highly cross-absorbed secondary antibody labelled with Alexa Fluor™ 647 (Invitrogen, #A21244) was diluted at 4 µg/ml. Cells were incubated with secondary antibodies for 45 min at room temperature. The FACSVerse instrument and the FACSSuite software (both from BD Biosciences) were used to acquire the data. FlowJo™ v10.8.1 Software (BD Life Sciences) was used to analyze the results.

### Caco-2 cell differentiation and exposure to chemotherapy agents

For Caco-2 cell differentiation to enterocyte-like cells, 0.12 × 10^6^ cells were seeded into 24-well plates containing polyethylene membrane inserts with a pore size of 0.4 μm and a culture area of 0.33 cm^2^ (Stem Cell Technologies). Cells were differentiated for 15 days, changing medium every 3–4 days. RA was always added during the differentiation at a concentration of 1.15 µg/ml unless specified differently. Differentiated cells were then exposed to 50 µg/ml of 5 FU or 1 µg/ml of Doxo on the apical and basolateral sides or were left unexposed as a control. Following incubation for 24 h, cells were washed twice with sterile PBS and were left to rest for 0–72 h. The integrity of cell monolayers was evaluated by taking the transepithelial electrical resistance (TEER) measurements and by using FITC-dextran permeability assay. TEER values were measured before and 5 FU exposure (prior cell collection) using the EVOM voltammeter (World Precision Instruments). Each value is the average of two to four independent measurements. The final values of the blank wells (containing no cells) were subtracted from each well. The final unit area (Ω cm^2^) was calculated by multiplying the TEER values with the surface area of the insert. Cells with the TEER greater than 500 Ω.cm^2^ were considered as fully differentiated Caco-2 cells. Cellular permeability was measured using fluorescein isothiocyanate (FITC) dextran FD4 (Sigma Aldrich). Prior FITC-dextran addition, cells were resuspended in DMEM without phenol red. A total of 1 mg/ml of FITC-dextran was added to the apical side of the wells for 2 h and the plates were incubated at 37 °C in the dark. Following incubation, the supernatant from the basolateral side of the wells was transferred into standard clear 96-well plates in triplicates. The fluorescence was measured using multi-mode microplate reader Spectramax i3Xn (Molecular Devices) at a 490 nm excitation and 520 nm emission wavelengths. The apparent permeability coefficient (Papp) was calculated as follows; Papp=(dQ/dt)x(1/AC_0_), where dQ/dt was the rate of FD4 appearance on the basolateral side divided by the incubation time, A was the surface area of the monolayer and C_0_ was the initial FD4 concentration (µg/ml). The final values of the blank wells (only containing cell culture medium) were subtracted from each sample. The samples were normalized to the wells containing 5 FU only exposed cells.

### Differentiated Caco-2 cells stimulation with soluble bacterial components

A total of 0.12 × 10^6^ cells were seeded into 24-well plates containing polyethylene membrane inserts and were left to attach for 4 days. Then half of the cells were differentiated in the presence of LR bacterial components, including 10% CFS, 100 µg/ml of EPS or 5 × 10^7^/ml of MV (prior-chemotherapy conditions). After 10 days, cells were exposed to 50 µg/ml of 5 FU on the apical and basolateral sides or were left unexposed as a control. Following 24 h incubation with 5 FU, cells were washed twice with PBS and then were resuspended in fresh medium with or without LR bacterial components, including 10% CFS, 100 µg/ml of EPS or 5 × 10^7^/ml of MV (post-chemotherapy conditions). After 72 h, cells and supernatants were collected for downstream applications. The concentration of EPS used in this study was chosen based on previously published work^15–17^.

### Primary small intestinal epithelial cell culture

Human primary small intestinal epithelial cells (DBFF-1123-HX977) were expanded in T25 flask pre-coated with gelatin-based coating solution (DBFF-1123-HX977C) using epithelial cell complete medium (DBFF-1123-HX977M) according to the instructions from the manufacturer (Creative Biolabs). Cells were detached using 0.25% Trypsin-EDTA (1X) solution (Gibco™) once the confluency reached approximately 90%. For differentiation, 0.07 × 10^6^ cells were seeded into 24-well plates containing polyethylene membrane inserts with a pore size of 0.4 μm and a culture area of 0.33 cm^2^ (Stem Cell Technologies). The cells were submerged in 200 µl of complete epithelial cell medium on the apical side and 500 µl on the basolateral side for 2 days until they became fully confluent. Then, the cells were kept in the air-liquid interface (ALI) culture conditions throughout the whole differentiation period of 21 days. Medium on the basolateral side was replenished every 2–3 days. On day 21, the cells were exposed to 5 FU at a concentration of 50 µg/ml on the basolateral side using 500 µl/well, and at a concentration of 100 µg/ml on the apical side using 25 µl/insert. After 24 h, the cells were carefully washed twice with sterile PBS. Then EPS was added on the apical side at a concentration of 100 µg/ml using 25 µl/insert. The cells were stimulated with EPS every 24 h for 3 days. The TEER values were measured on day 2 (before air-lifting), days 7, 14, 21 and at the end of the cell culture (prior permeability assay). The permeability was measured using FITC-dextran as described above.

### Macrophages polarization with Caco-2 cell supernatant and immunophenotyping

Peripheral blood mononuclear cells (PBMC) were isolated from buffy coats using Ficoll-Hypaque (Cytiva) gradient centrifugation, and monocytes were isolated from PBMC by negative selection using EasySep™ human monocyte enrichment kit (STEMCELL Technologies), according to the manufacturer’s instructions. Monocytes were seeded into 48-well plates at 1 × 10^6^/ml concentration using 0.5 ml/well DMEM complete medium and were left to attach for 2 h. Afterwards, the medium was removed and the supernatant from Caco-2 cells was added instead. Monocytes were left to differentiate for 6 days, and then LPS was added at 100 ng/ml for 24 h. Matured macrophages were detached using macrophages detachment medium (PromoCell) and then were transferred to 96-well plates for staining. The fixable viability stain 780 (BD Biosciences) or the Live/Dead Fixable Dead Cell Stain Kit-Aqua (Life Technologies) was used to exclude non-viable cells. Macrophages were stained with alternating combinations of antibodies including; CD14 FITC (clone M5E2) (BD Biosciences), HLA-DR PerCP (clone L243), HLA-DR APC-Cy7 (clone L243), CD206 PerCP-Cy5.5 (clone 15 − 2), CD163 APC (clone GHI/61), CD80 PE-Cy7 (clone 2D10), CD86 PE (clone 2331, FUN-1) and PD-L1 BV421 (clone MIH1) (all from BioLegend). After extracellular staining, cells were either washed and fixed using 4% paraformaldehyde (Sigma Aldrich), or they were fixed and permeabilized using Fix/Perm Buffer (BD Biosciences) for intracellular staining according to the instructions from the manufacturer. Then cells were stained with titrated amount of intracellular CD68 PE antibody (clone Y1/82A) (BD Biosciences). The data were acquired using FACSVerse instrument and the FACSSuite Software (both from BD Biosciences). FlowJo Software (TreeStar) was used to analyze the results.

### RNA sequencing

Differentiated Caco-2 cells were exposed to 50 µg/ml of 5 FU on the apical and basolateral sides or were left unexposed as a control. After 24 h incubation, cells were washed twice with PBS and then were resuspended in fresh medium with or without LR bacterial components, including either 10% CFS, 100 µg/ml of EPS or 5 × 10^7^/ml of MV (post-chemotherapy conditions). Cells were incubated for 72 h and then were resuspended in elution buffer (Thermo Fisher Scientific). Total RNA was extracted using mirVana isolation kit according to the manufacturer’s instructions (Thermo Fisher Scientific).

RNA sequencing was performed at the core facility for Bioinformatics and Expression Analysis (BEA), and the resulting count matrix was obtained for downstream analysis. Normalization and differential expression analysis were conducted using the DESeq2 package in R (version 4.3.3). Genes with a fold change greater than 1.5 (log2FC > 0.58 or < -0.58) and an adjusted p-value < 0.05 were considered significantly differentially expressed. Volcano plots were created using the ggplot2 package to highlight significantly upregulated and downregulated genes. Gene Ontology (GO) enrichment analysis was performed using the enrichGO function from the clusterProfiler package, focusing on significantly altered genes from each comparison to identify overrepresented biological processes and molecular functions.

### Enzyme-linked immunosorbent assay

The levels of CXCL8/IL-8 and TGFβ1 (all from MabTech AB), as well as CCL20 (R&D systems, Bio-Techne) were measured in the cell culture supernatant of Caco-2 cells according to the manufacturer’s instructions. Soluble TNF-a, IL-6, IL-1b and IL-10 (all from MabTech AB), as well as IL-1ra and CD163 (R&D systems, Bio-Techne) were measured in the cell culture supernatant of macrophages on Day 7 according to the manufacturer’s instructions. The results were analysed using SoftMax Pro 5.2 rev C (Molecular Devices Corp.).

### Statistical analysis

**Fig. 1:**
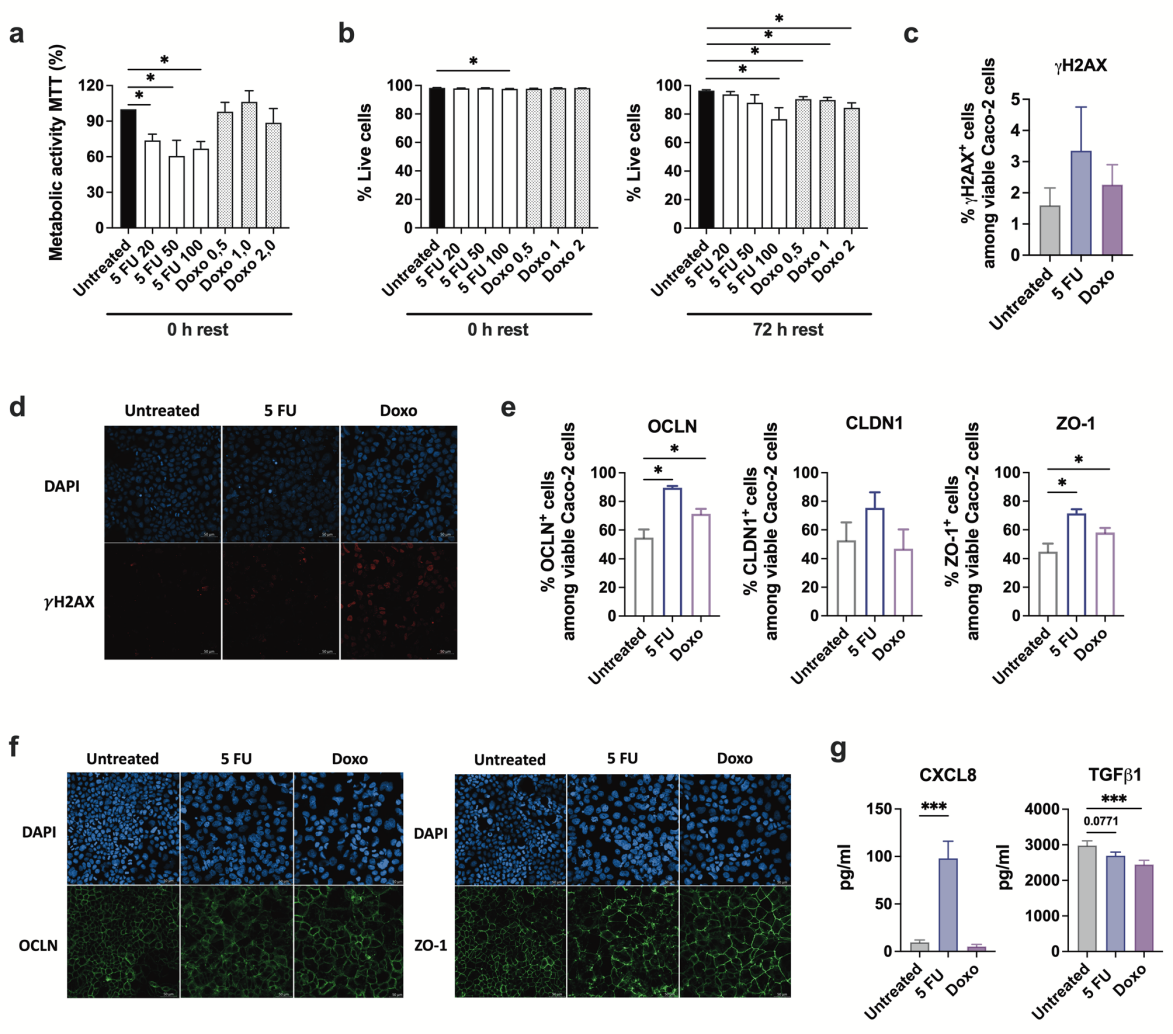
The effects of chemotherapy drugs on the metabolic activity, viability, barrier integrity, and functional response in Caco-2 cells. The cells were exposed to 50 µg/ml of 5 FU or 1 µg/ml of Doxo for 24 h, and then were rested for additional 24 h unless stated otherwise. **(a)** The metabolic activity in Caco-2 cells following exposure to increasing concentrations of 5 FU or Doxo. **(b)** The percentage of viable Caco-2 cells assessed by flow cytometry immediately after chemotherapy exposure, or 72 h after the drugs were washed away. **(c)** The percentage of γH2AX^+^ Caco-2 cells measured by flow cytometry, and **(d)** immunofluorescent staining of γH2AX marker. **(e)** The percentage of OCLN, CLDN1 and ZO-1 expressing viable Caco-2 cells measured by flow cytometry. **(f)** The evaluation of tight junction protein OCLN and ZO-1 expression using immunofluorescent staining. **(g)** Soluble CXCL8 and TGFβ1 levels in the culture supernatant of chemotherapy exposed Caco-2 cells. Results are presented as mean ± SEM from three independent experiments (*n* = 3–12). Wilcoxon matched-pairs signed rank test was applied to determine statistical differences, *p < 0.05, **p < 0.01, ***p < 0.001. The images were taken at 20x magnification.

**Fig. 2:**
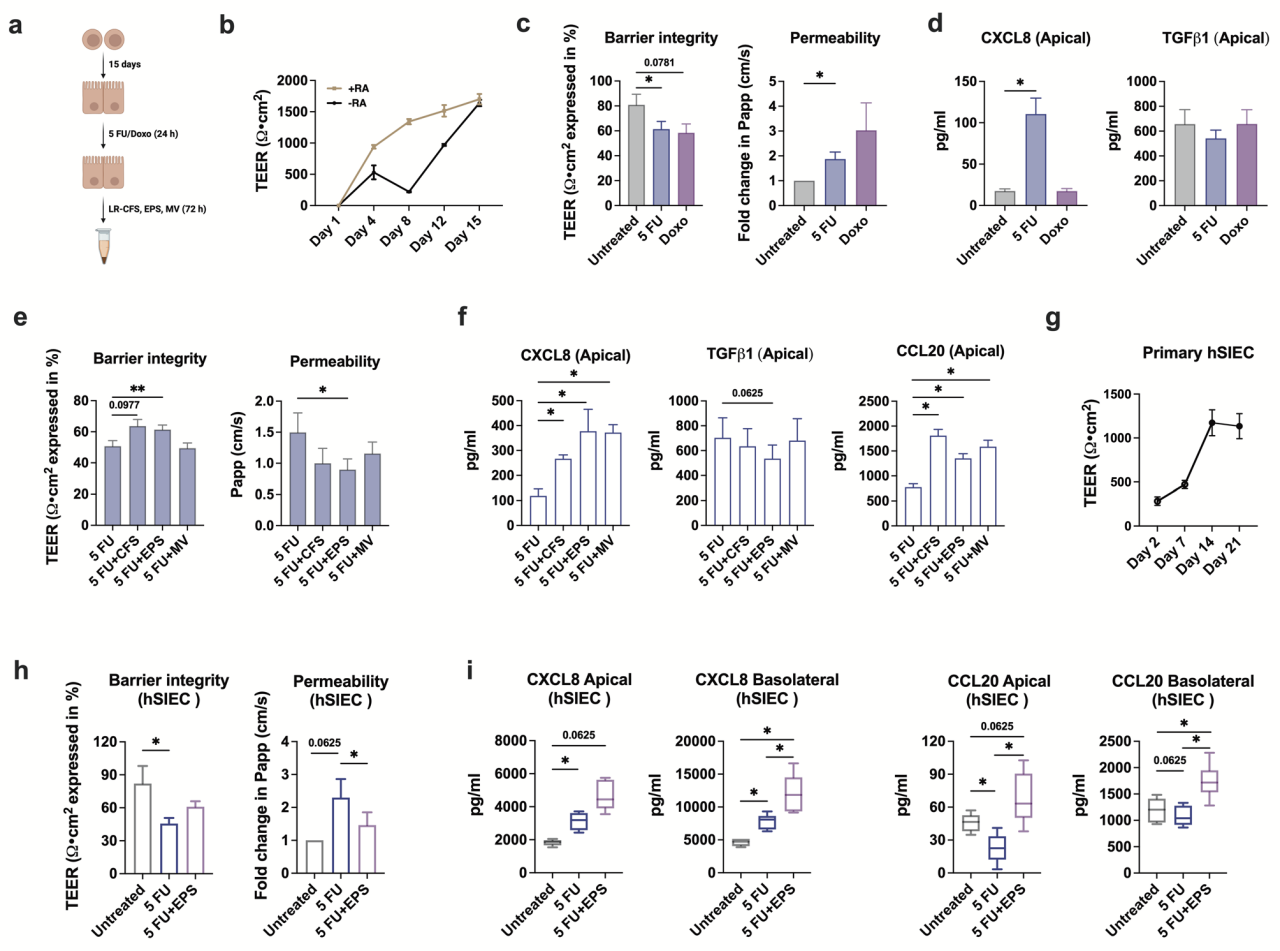
LR-derived EPS improve IEC barrier integrity after 5 FU exposure. Caco-2 cells were differentiated for 14 days and then were exposed to 50 µg/ml of 5 FU or 1 µg/ml of Doxo for 24 h. Following 5 FU removal, the cells were cultured with bacterial components for 72 h. **(a)** Experimental model of Caco-2 cell culture. **(b)** The transepithelial electrical resistance (TEER) values, expressed as Ωcm^2^, measured in Caco-2 cells differentiated with or without RA. TEER values were corrected for the blank wells. **(c)** The TEER values and permeability, measured by FITC-dextran transport at cm/s, in differentiated Caco-2 cells upon 5 FU or Doxo exposure. Permeability was normalized to the control cells that were not exposed to chemotherapy drugs. **(d)** Soluble levels of CXCL8 and TGFβ1 in the culture supernatant of Caco-2 cells taken from the apical part of the insert following exposure to 5 FU or Doxo. **(e)** The TEER values and permeability in Caco-2 cells stimulated with LR-CFS, EPS or MV after 5 FU exposure. **(f)** Apical CXCL8, TGFβ1 and CCL20 secretion by Caco-2 cells stimulated with bacterial components after 5 FU exposure. **(g)** TEER values measured in hSIEC throughout the differentiation period of 21 days. **(h)** TEER values and permeability of hSIEC with or without EPS stimulation following 5 FU exposure. The permeability was normalized to the control cells that were not exposed to chemotherapy drugs. **(i)** Apical as well as basolateral secretion of CXCL8 and CCL20 from hSIEC. In (c, e, h) TEER values were corrected for the blank wells and then were expressed as percentage relative to the TEER values prior chemotherapy. (b) Results are presented as mean ± SEM from one independent experiment (*n* = 4). (**c**–**i**) Results are presented as mean ± SEM from two to four independent experiments (*n* = 5–8). Wilcoxon matched-pairs signed rank test was applied to determine statistical differences, *p < 0.05, **p < 0.01.

The statistical analysis was performed using GraphPad Prism 8 (GraphPad Prism Inc.). Paired Friedman test followed by Dunn´s multiple comparison or Wilcoxon matched-pairs signed rank test was applied to determine the differences between the groups (Figs. 1, 2, 4 and 5).

P-values < 0.05 were considered statistically significant.

## Results

### FU impairs viability and barrier integrity, and mediates an inflammatory response in Caco-2 cells

To examine how 5 FU affects the growth and proliferation of IEC, we first exposed Caco-2 cells to increasing concentrations of 5 FU for 24 h, then measured metabolic changes using MTT assay and assessed viability using flow cytometry. To compare 5 FU effects to another chemotherapy agent with different mechanisms of action, we also included Doxo, which is an anthracycline antibiotic widely used for hematologic cancer treatment^18^. The concentration of both drugs was chosen based on the previously published data^19,20^. The metabolic activity of Caco-2 cells significantly decreased upon 5 FU exposure regardless of concentration, whereas Doxo did not markedly affect the cells at any dose tested (Fig. 1a). On the other hand, both 5 FU and Doxo reduced the cell viability in a dose dependent manner, especially after 72 h recovery period (Fig. 1b). In light of the above, 50 µg/ml of 5 FU and 1 µg/ml of Doxo were chosen for the following experiments. We next investigated γH2AX (a marker for early DNA damage) in Caco-2 cells upon chemotherapy agent addition for 24 h. The percentage of cells positive for γH2AX was higher in 5 FU and Doxo treated cells compared to the control, although not statistically significant (Fig. 1c). Immunofluorescent staining of γH2AX showed increased staining upon both 5 FU and Doxo treatment (Fig. 1d). To assess the barrier integrity of cells, we measured the expression of TJP including transmembrane proteins OCLN and CLDN1, and cytosolic protein ZO-1 (also known as TJP-1). Flow cytometric analyses showed a significant increase in the frequency of OCLN- and ZO-1-positive cells upon 5 FU and Doxo exposure, while no significant change in the CLDN1 expression was noted (Fig. 1e). Immunofluorescent staining of OCLN and ZO-1 showed no significant increase in signal intensity, however a clear disruption of the characteristic web-like expression pattern with increased cytoplasmic localization upon chemotherapy addition was observed for both TJP (Fig. 1f). Finally, secretion of the chemokine CXCL8 was significantly increased upon 5 FU exposure, while the cytokine TGFβ1 was markedly lower in response to both, 5 FU and Doxo (Fig. 1g).

### LR-derived EPS improve IEC barrier integrity, despite of enhanced CXCL8 and CCL20 levels upon 5 FU exposure

Next, Caco-2 cells were differentiated into a monolayer with small-intestinal enterocyte-like morphology and function. Given that retinoids, including retinoic acid (RA), contribute to intestinal barrier formation in vivo^21^, polarization was performed in the presence of RA. Selective probiotic bacteria, like LR, have been shown to modulate chemotherapy-induced toxicity in vivo and in vitro^6,9^, therefore, we examined if LR-derived metabolites would alter chemotherapy-induced effects. The secreted components from LR, including CFS, EPS and MV, were added for 3 days after 5 FU was removed (Fig. 2a).

First, transepithelial electrical resistance (TEER) was measured to evaluate intestinal barrier integrity in differentiated Caco-2 cells, which increased more steadily when cells were cultured with RA (Fig. 2b). Secondly, both 5 FU and Doxo reduced TEER and increased the apical to basolateral translocation of FITC-labelled dextran (Fig. 2c). Finally, apically secreted CXCL8 levels were significantly upregulated following 5 FU, but not Doxo exposure, while TGFβ1 did not differ among the groups (Fig. 2d). Basolateral levels of CXCL8 and TGFβ1 were low and did not differ among the groups (Supplemental Fig. 1a). Based on its potent inflammatory-inducing properties, 5 FU was used in all subsequent experiments.

When secreted LR components were added to cells after 5 FU exposure, TEER was significantly increased and the translocation of FITC-labelled dextran was reduced by EPS. Similar effects were observed when stimulating with CFS, although not to a significant level, while addition of MV had no effect compared to cells only exposed to 5 FU (Fig. 2e). Further, apical secretion of CXCL8 and CCL20 was significantly enhanced by CFS, EPS and MV stimulation compared to the cells exposed to 5 FU alone (Fig. 2f). Apical TGFβ1 level did not significantly differ among the groups; however, the secretion was markedly reduced upon stimulation with EPS (Fig. 2f). CXCL8 and TGFβ1 secretion from the basolateral side was markedly lower compared to the apical side and did not differ among the groups, whereas basolateral CCL20 was significantly upregulated after cells were stimulated with CFS and MV, although overall levels were also lower compared to the apical side (Supplemental Fig. 1b).

Additionally, we differentiated Caco-2 cells in the presence of CFS, EPS and MV, and then exposed them to 5 FU to assess if bacterial components stimulation prior to chemotherapy could protect the cells against the damage later. The TEER values and permeability of Caco-2 cells were not improved by any bacterial components when they were added during cell differentiation (Supplemental Fig. 2a). Soluble CXCL8 and CCL20 levels were comparable among different treatment conditions from both apical and basolateral sides, except apical TGFβ1 secretion which was lower after 5 FU exposure in cells differentiated in the presence of CFS and MV compared to unstimulated cells (Supplemental Fig. 2b). Of note, we observed similar results in undifferentiated Caco-2 cells when CFS was introduced prior to or post-chemotherapy exposure. Soluble levels of CXCL8, TGFβ1 and CCL20 were comparable among the groups when CFS was added prior to 5 FU exposure, however, CXCL8 and CCL20 were significantly upregulated when CFS was added post-5 FU exposure (Supplemental Fig. 3).

To corroborate our findings in a model that better approximates the *in vivo* intestinal environment, we differentiated primary hSIEC from a healthy adult donor, exposed them to 5 FU and then stimulated with EPS. The TEER in differentiated hSIEC were similar to those measured in Caco-2 cells (Fig. 2g). The exposure to 5 FU significantly reduced TEER and increased translocation of FITC-labelled dextran, while stimulation with EPS after 5 FU exposure significantly reduced translocation of FITC-labelled dextran and tended to increase TEER (Fig. 2h). Significantly higher apical and basolateral levels of CXCL8 and CCL20 were measured in the culture supernatant of EPS stimulated hSIEC compared to 5 FU only exposed cells (Fig. 2i). TGFβ1 was only secreted from the apical side and did not differ among the groups (Supplemental Fig. 4).

### LR-derived EPS alter the transcriptional program associated with intestinal barrier maintenance

**Fig. 3:**
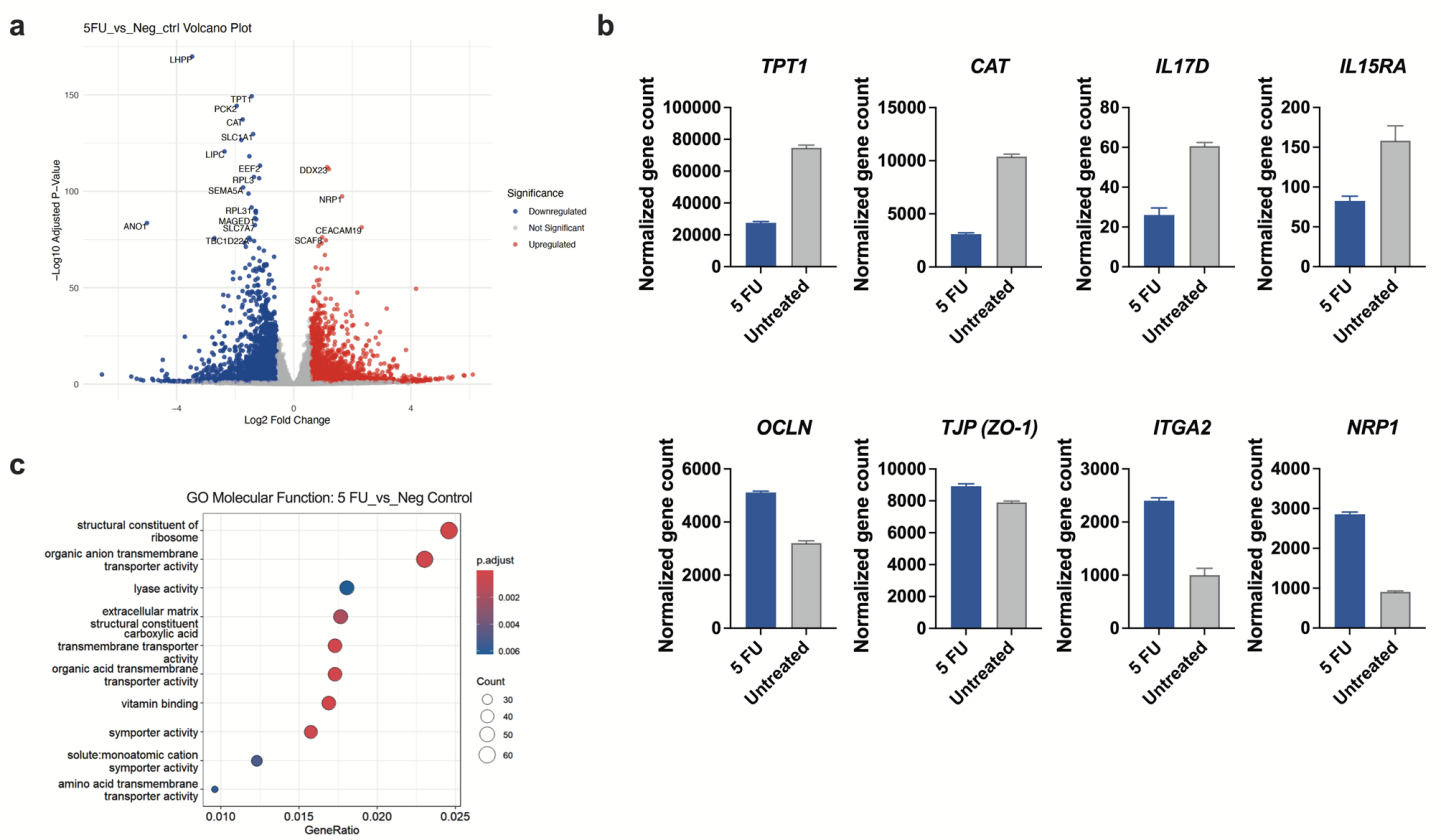
Transcriptional changes in 5 FU-exposed Caco-2 cells. Differentiated Caco-2 cells were exposed to 50 µg/ml of 5 FU for 24 h. Following 5 FU removal, the cells were cultured for 72 h and then were collected for non-targeted RNA-seq analysis. **(a)** A volcano plot showing gene expression in Caco-2 cells exposed to 5 FU versus unexposed cells. Genes with a fold change greater than 1.5 (log2FC > 0.58 or < -0.58) and an adjusted p-value < 0.05 were considered significantly differentially expressed. Volcano plots were created using the ggplot2 package to highlight significantly upregulated (in red) and downregulated (in blue) genes. **(b)** Normalized gene counts for *TPT1*, *CAT*, *IL17D*, *IL15RA*, *OCLN*, *TJP* (ZO-1), *ITGA2* and *NRP1*. **(c)** Gene Ontology (GO) enrichment analysis of molecular functions associated with significantly expressed genes in 5 FU exposed versus unexposed Caco-2 cells. The *x*-axis represents the gene ratio, and the y-axis lists the enriched GO terms ranked by their significance. The size of the points reflects the number of genes mapped to each term, and the color gradient indicates the statistical significance based on the adjusted p-values. (b) Results are presented as mean ± SEM from independent experiments (*n* = 3).

To further explore the intracellular signalling cascade upon LR stimulation of 5 FU exposed cells, a comprehensive analysis of the transcriptional program in Caco-2 cells was performed using non-targeted RNA-seq. Firstly, many genes were altered upon 5 FU exposure alone and the most differentially expressed genes (DEGs) are highlighted in the volcano plot (Fig. 3a). Among the DEGs, 5 FU significantly downregulated genes such as *TPT1* and *CAT*, which encode proteins associated with cell survival^22^ and antioxidant defence^23^ respectively, but also genes such as *IL17D* and *IL15RA*, which regulate immune responses at the mucosal site^24,25^ (Fig. 3b, upper graphs). On the contrary, 5 FU significantly increased the expression of genes encoding tight junctions such as OCLN and ZO-1, integrin α2β1, and membrane-bound protein neuropilin-1 known as a coreceptor for TGFβ1 (Fig. 3b, lower graphs). To identify molecular functions associated with the DEGs, we also performed GO enrichment analysis. GO terms such as structural constituent of ribosome and transmembrane transporter activity were among the most significantly enriched (Fig. 3c).

**Fig. 4:**
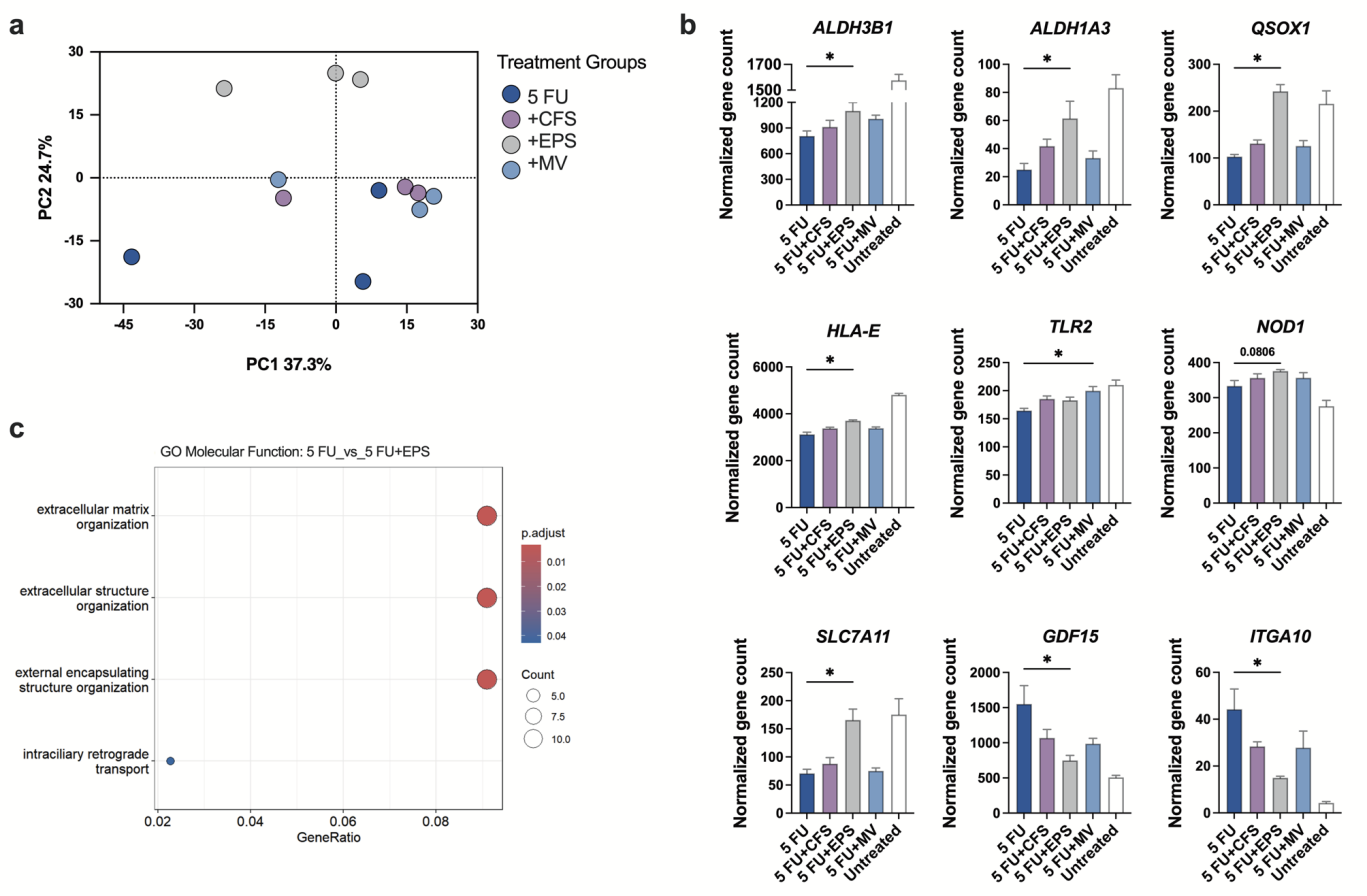
LR-derived EPS alter the transcriptional program associated with intestinal barrier maintenance. Differentiated Caco-2 cells were exposed to 50 µg/ml of 5 FU for 24 h. After 5 FU removal, the cells were cultured with bacterial components for 72 h and then were collected for RNA-seq analysis. **(a)** Principal component analysis (PCA) plot includes the log transformed data of 1000 most variable genes among cells exposed to 5 FU alone and cells stimulated with CFS, EPS or MV after 5 FU exposure. The percentage of variance explained by the principal components (PC) 1 and 2 are indicated on the axis. **(b)** Normalized gene counts for *ALDH3B1*, *ALDH1A3*, *QSOX1*, *HLA-E*, *TLR2*, *NOD1*, *SLC7A11*, *GDF15* and *ITGA10* obtained from RNA-seq results. **(c)** Gene Ontology (GO) analysis of molecular functions associated with all significantly expressed genes in 5 FU exposed versus 5 FU exposed and EPS stimulated cells. The *x*-axis represents the gene ratio, and the y-axis lists the enriched GO terms ranked by their significance. The size of the points reflects the number of genes mapped to each term, and the color gradient indicates the statistical significance based on the adjusted p-values. (b) Results are presented as mean ± SEM from two independent experiments (*n* = 3). Paired Friedman test followed by Dunn´s multiple comparison was used to determine statistical difference. The 5 FU unexposed cells were not included in the statistical analysis to focus on the effects of bacterial components after 5 FU exposure, *p < 0.05.

**Fig. 5:**
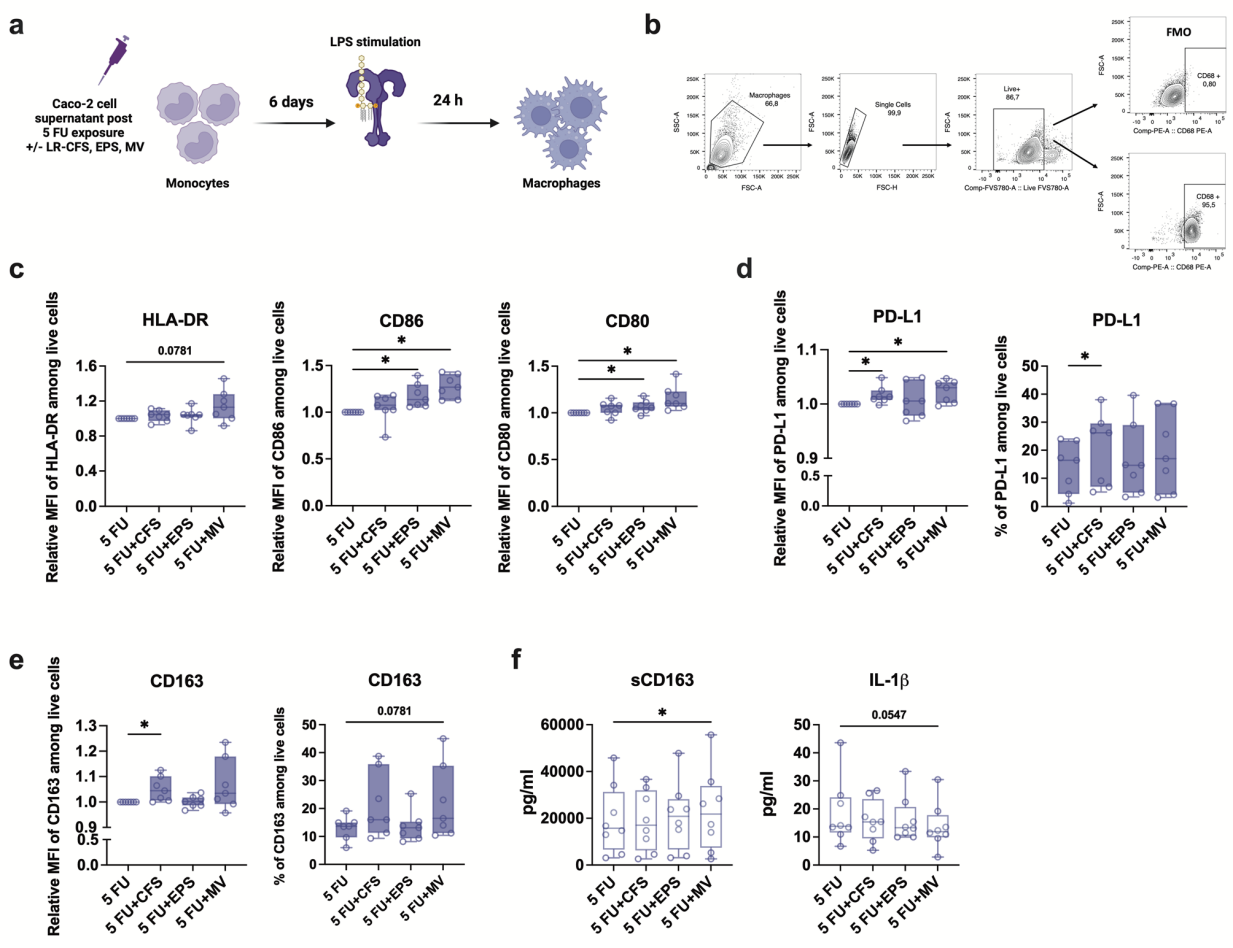
EPS stimulation of 5 FU exposed Caco-2 cells drives M1-like macrophages polarization. Monocytes were isolated and cultured for 6 days in the presence of 5 FU exposed Caco-2 cell supernatant stimulated with either CFS, EPS or MV. On day 6, macrophages were activated with LPS for 24 h. **(a)** Experimental model of macrophages polarization. **(b)** Representative flow cytometry analysis of CD68 expression in viable macrophages on day 7. **(c)** The relative mean fluorescent intensity (MFI) of HLA-DR, CD86 and CD80 expression on macrophages among all donors. **(d)** The relative MFI of PD-L1 and the percentage of PD-L1 expressing macrophages among all donors. **(e)** The relative MFI of CD163 and the percentage of CD163 expressing macrophages among all donors. **(f)** Soluble CD163 and IL-1β levels in the culture supernatant of macrophages. MFI results were normalized to the values obtained from cells exposed to 5 FU only. Results are presented as boxplots from three independent experiments, displaying the range from minimum to maximum values including all data points (*n* = 7–8). Wilcoxon matched-pairs signed rank test was applied to determine statistical differences, *p < 0.05.

To compare transcriptional patterns among cells stimulated with CFS, EPS and MV after 5 FU exposure, we conducted principal component analysis (PCA) including 1000 gene expression variables. Cells stimulated with EPS clearly clustered away from all other stimulation groups and with cells exposed solely to 5 FU (Fig. 4a and Supplemental Fig. 5). Notably, EPS modulated a broad set of genes related to stress responses, epithelial structure, and innate immune pathways. Among many others, *ALDH3B1*, *ALDH1A3*, *QSOX1*, *HLA-E*, and *SLC7A11* expression was significantly upregulated, while *GDF15* and *ITGA10* were downregulated by EPS addition compared to 5 FU alone (Fig. 4b). The expression of pattern recognition receptor (PRR) *NOD1* tended to be upregulated by EPS stimulation, while *TLR2* was significantly higher by MV addition (Fig. 4b). Other genes that clearly differed between EPS stimulated cells and the rest of the groups are included in the (Supplemental Fig. 6a). Molecular functions associated with all DEGs from 5 FU exposed and EPS stimulated cells versus 5 FU exposed cells included extracellular matrix and structure organization (Fig. 4c), while top biological processes associated with the DEGs also comprised of RA biosynthesis and Basigin interaction GO terms (Supplemental Fig. 6b).

### Monocyte culture with the supernatant from 5 FU exposed and EPS stimulated Caco-2 cells induces M1-like macrophage polarization

A crosstalk between IEC and immune cells in the gut is crucial for adequate intestinal homeostasis. Therefore, we assessed if CFS, EPS and MV stimulation after 5 FU exposure of Caco-2 cells also had an impact on immune cells. Freshly isolated monocytes were cultured in the presence of conditioned media from 5 FU exposed and stimulated Caco-2 cells for 6 days and then immunophenotyped LPS-activated macrophages using flow cytometry (Fig. 5a).

To confirm successful differentiation into macrophages in the presence of conditioned Caco-2 medium, cells were analysed for CD68 expression. Indeed, more than 80% of cells expressed CD68 upon LPS activation at the end of culturing with conditioned Caco-2 medium (Fig. 5b). Next, we assessed the expression of CD80, CD86 and HLA-DR – key characteristics by M1-like (classically activated) macrophages, and CD163, CD206 and PD-L1 which are expressed on M2-like (alternatively activated) macrophages. As expected, the percentages of HLA-DR^+^ and CD86^+^ macrophages were high and did not differ significantly among the groups (Supplemental Fig. 6a). However, the expression level of CD86, measured as mean fluorescent intensity (MFI), was significantly upregulated when monocytes were cultured with the conditioned media from EPS or MV stimulated Caco-2 cells compared to the cells only exposed to 5 FU (Fig. 5c). The expression of CD80 was also significantly higher on these macrophages (Fig. 5c), and similarly, the percentage of CD80^+^ macrophages increased under these conditions (Supplemental Fig. 7a). However, conditioned media from MV stimulated Caco-2 cells also significantly increased the expression of M2-like-associated marker PD-L1 on differentiated macrophages (Fig. 5d). Differentiation of macrophages in the presence of conditioned media from CFS stimulated Caco-2 cells after 5 FU exposure resulted in significantly increased frequency of PD-L1^+^ cells (Fig. 5d) and increased expression levels of both PD-L1 and CD163 compared to control cells (Fig. 5d, e).

Finally, we investigated the functional response of these macrophages after LPS activation by quantifying secreted cytokines in the culture supernatants. Conditioned media from MV stimulated Caco-2 cells induced significant secretion of soluble CD163 and inhibited the secretion of IL-1β (Fig. 5f). Secretion of the cytokines TNF-α, IL-6, IL-10 and IL-1 receptor antagonist (ra) were abundant in all samples and did not differ between any groups of conditioned media compared with control cells (Supplemental Fig. 7b).

## Discussion

Chemotherapy-induced gastrointestinal toxicity is a dose-limiting toxicity of cancer treatment, which often results in severe complications compromising remission and treatment success for patients^1,2,26^. The potential of probiotics in alleviating therapy-mediated cytotoxicity has been proposed in several studies^5,6,27^, however, safety prospectives should be considered to avoid systemic infections using viable bacteria in immunocompromised patients. The detrimental effects of probiotic pre-treatment were also described in a mouse model of *Pseudomonas aeruginosa* sepsis induced by chemotherapy^28^, indicating that live probiotic bacteria may not always be effective in immunosuppressed conditions. Here, we demonstrate that EPS, a bioactive compound from probiotic LR bacteria, is favourable for the recovery of chemotherapy-impaired intestinal barrier damage due to modulation of genes associated with extracellular matrix organization and RA signalling axis, and induction of immune responses important for the early phases of tissue repair.

Chemotherapy agents have a diverse range of mechanisms to target different pathways involved in cancer pathogenesis. Indeed, we found that the exposure to 5 FU, but not Doxo, decreased Caco-2 cell metabolic activity, while the viability was affected by both drugs. It is known that 5 FU directly impairs nucleic acid synthesis and thus have a profound effect on cellular metabolism^29^. Doxo is the anthracycline antibiotic which intercalates DNA, contributes to free radical formation, and has direct effects on lipid peroxidation with a secondary impact on cellular metabolism^18^. The barrier integrity of Caco-2 cells was compromised upon treatment with 5 FU and Doxo, as evident from decreased TEER with a concomitant increase in translocation of FITC-labelled dextran. Although our flow cytometry results showed a higher percentage of OCLN and ZO-1 positive cells it is important to consider that flow cytometry measures protein presence at the single-cell level but does not provide spatial information on junctional organization. A plausible explanation is that tight junction proteins are upregulated or retained in mislocalized or intracellular pools as a compensatory response yet fail to assemble into fully functional junctions. This may indicate collapsed TJ that fail to maintain cell-cell junctions, where the increased relative expression could represent a compensatory mechanism or stress-induced upregulation. Thus, the apparent discrepancy reflects differences between expression and organization, rather than conflicting results. Previous studies have indeed shown that reactive oxygen species can disrupt the organization of actin cytoskeleton (reviewed^30^. Both OCLN and ZO-1 are associated with the cytoskeleton fraction of the cell, and such disruption may impair the trafficking and localization of tight junction components, leading to accumulation of non-functional or misassembled proteins despite increased expression levels. We also cannot rule out the possibility that fluctuations in TJP expression could be a chemotherapy dose-dependent response. This has previously been reported in mice injected with 5 FU, where a lower dose mediated OCLN and CLDN1 expression, while a higher dose significantly reduced the production of both TJP^31^. We also found that 5 FU altered the expression of genes associated with cell survival and antioxidant defence, which is not unexpected, as chemotherapy drugs induce the production of reactive oxygen species that might overwhelm antioxidant system resulting in related genes downregulation^32^.

LR exerts beneficial properties through various molecules including but not limited to; antimicrobial compounds, lactic acid, peptidoglycans, different cell wall proteins, adenosine, EPS and MV, although the exact immunomodulatory effects are strain-specific^33–35^. We have recently reported that extracellular MV isolated from LR strains DSM 17938 and BG-R46^®^ carry TLR2 ligand lipoteichoic acid (LTA), contain bacterial surface proteins, RNA, DNA, and can modulate functional responses in immune and non-immune cells^34,36^. EPS are carbohydrate polymers either attached to the cellular surface or secreted into the surrounding milieu. EPS exhibit diverse biological activities including antioxidant-, anticancer-, antimicrobial-, and immunomodulatory properties^37^, and they activate different PRRs depending on the cell type and acidity nature of the EPS^38^. Here we found that EPS, but not MV, facilitated the repair of epithelial barrier integrity after chemotherapy exposure. Our transcriptomic analysis identified several candidate pathways that may underlie the beneficial effects of EPS on epithelial barrier repair, including extracellular matrix organization and extracellular structure organization, external encapsulating structure organization, and RA biosynthesis. For instance, EPS significantly upregulated the expression of the gene encoding ALDH3B1, which detoxifies reactive aldehydes generated during oxidative stress and thus protects cells from damage^39^. *ALDH1A3* was also induced by EPS, suggesting the activation of RA detoxification pathways that may promote epithelial differentiation and contribute to the restoration of barrier integrity^40^. The beneficial effects of RA on intestinal barrier maintenance have been demonstrated in multiple studies, which show that RA enhances epithelial differentiation and tight junction formation while mitigating cytokine-induced barrier disruption in intestinal epithelial models^41,42^. Since RA was present in the culture medium, the upregulation of RA biosynthesis-associated genes by EPS may indicate that the latter potentiates RA-mediated differentiation and repair programs, thereby contributing to the accelerated restoration of barrier integrity observed in our model. Further, EPS-induced *SLC7A11* encodes cystine/glutamate antiporter which plays a key role in the antioxidant defence^43^, while *MMP1* encodes matrix metallopeptidase-1 necessary for collagen degradation enabling tissue remodelling during physiological and inflammatory conditions^44^. Among others, EPS upregulated *QSOX1* expression, which has been shown to maintain colon mucosal barrier^45^, and downregulated *GDF15* inflammatory gene expression, which is often induced under stress conditions^46^. Future studies should focus on targeted functional validation of the RA biosynthesis and ECM remodelling pathways to determine which plays the predominant role in EPS-mediated epithelial repair. For example, inhibition of key ECM-modifying enzymes, integrin-mediated signalling, or use of RA receptor antagonists, would reveal whether EPS-induced barrier restoration is attenuated.

We further found that monocyte cultured in the presence of conditioned cell media from 5 FU exposed and EPS stimulated Caco-2 cells significantly upregulated M1-like macrophages markers. Based on functional characteristics, M1-like macrophages are known as inflammatory cells crucial for tissue clearance and host defence. However, they have been also reported to increase colon epithelial cell proliferation and renewal through their soluble factors, in contrast to M2-like macrophages that require physical contact with the colon epithelium^47^. Of note, in our study macrophages were exposed to conditioned medium from EPS-treated epithelial cells rather than EPS directly, indicating that the observed polarization likely arises as a downstream consequence of the chemokine and cytokine rich environment generated by EPS-stimulated IECs. Macrophages are well known to play a dual role in tissue repair, initially amplifying inflammation through the secretion of chemotactic factors and later transitioning into a pro-reparative phenotype that releases growth factors promoting cell proliferation, angiogenesis, and extracellular matrix remodelling. As repair progresses, macrophages adopt anti-inflammatory state that helps resolve inflammation (reviewed^48^. Future studies, employing cytokine/chemokine neutralization in the conditioned medium would help to determine which exact mediators causally drive this macrophage response. EPS from *Limosilactobacillus mucosae* CCFM1273 have been reported to lessen inflammation in a mouse model of inflammatory bowel disease (IBD) through several mechanisms such as restoration of goblet numbers, induction of intercellular junctions, and inhibition of apoptosis^49^. *Lacticaseibacillus paracasei* IJH-SONE68-derived EPS have been reported to prevent and alleviate inflammation in mice with IBD via upregulation of anti-inflammatory IL-10 cytokine, while pro-inflammatory response was reduced^50^. LR-derived EPS have been shown to modulate immune responses in a context dependent manner. Under pathological challenge with enterotoxigenic *Escherichia coli* (ETEC), EPS derived from LR DSM 17938 and L26 strains have been demonstrated to attenuate bacterial adhesion to porcine epithelial cells. However, pro-inflammatory IL-1β was significantly induced by DSM 17938, while TNF-α and IL-6 expression were enhanced by the L26 strain^51^. Under challenge with *Salmonella Typhimurium*, pre-treatment with EPS isolated from the L26 strain have been shown to reduce the pro-inflammatory response in porcine intestinal epithelial cells^52^. Another study demonstrated that LR DSM 17938-derived EPS significantly increased the expression of IL-1β, IL-6, IL-12, and IL-10 in porcine monocyte-derived dendritic cells, promoting their maturation and activation^53^. Collectively, these findings indicate that the immunomodulatory properties of EPS are species and strain-specific, however, the host environment and disease context also influence the outcome. Further, the formulation process of EPS is also of significant importance and certain methods during EPS preparations can be used to alter bacterial metabolic activity and improve probiotic efficacy^14^. In this study, we observed positive changes in the intestinal barrier recovery when EPS were added post-chemotherapy exposure. Our results from enterocyte-like Caco-2 cells were confirmed in primary hSIEC, indicating the robustness of our findings in a more physiologically representative model. However, we cannot disregard the potential beneficial effects of EPS prior to chemotherapy if an in vivo model was used.

LR-derived CFS contains a mixture of bioactive compounds that are released during their growth in culture^33^. Here, we found that CFS somewhat enhanced the integrity of Caco-2 cells when added after chemotherapy exposure, although the results were not statistically significant. This is reasonable given that CFS also contains EPS, however, most likely at a lower concentration. MV have been previously shown to strengthen the integrity of intestinal epithelium against ETEC in a dose-dependent manner^34^. We did not assess the effects of different MV concentrations, which could potentially influence the observed impact of MV on barrier integrity. Additionally, differences in study design are most likely imperative here, as in the present study, barrier integrity was studied in the context of 5-FU-induced inflammation to which the effects of *L. reuteri* DSM 17938-derived MV on barrier integrity has so far not been evaluated. In agreement with others, MV significantly upregulated the expression of *TLR2*, while intracellular receptor *NOD1* expression was more affected by EPS. Further, the supernatant from 5 FU exposed and MV stimulated Caco-2 cells induced monocytes to exhibit a mixed phenotype of M1-like and M2-like macrophages, while CFS clearly polarized cells with M2-like surface markers. We stimulated IEC with bacterial components from the apical side of the culture insert, thereby we cannot rule out the possibility that they passed through the epithelial barrier and were present in the supernatant directly affecting monocytes. Nevertheless, our data suggest that the immunomodulatory effects of different LR components are mediated through their activation of distinct signalling pathways, leading to varied immune responses.

Furthermore, we found that CFS, MV and EPS significantly potentiated the 5 FU-induced secretion of soluble CXCL8 and CCL20. Interestingly, the highest levels of CXCL8 and CCL20 were observed in parallel with the greatest barrier protection following EPS treatment, which subsequently caused an increase in primarily M1 associated markers on conditioned macrophages. This finding appears paradoxical as CXCL8, CCL20 and M1 macrophages are typically associated with inflammation. However, although macrophages were skewed towards an M1-like phenotype, functionally, we observed no increased secretion of inflammatory M1-associated cytokines such as IL-1β or TNF-α. Furthermore, both CXCL8 and CCL20 can also participate in epithelial repair and homeostasis, modulating the balance between tolerance and inflammation. The chemokine CXCL8 rapidly promotes neutrophil activation and influx within the intestinal mucosa which is fundamental for the clearance of pathogens and necrotic tissues during early stages of tissue repair^54^. The primary role of CCL20 is to regulate cell-mediated immunity against intracellular bacteria via chemotaxis of CCR6-expressing adaptive immune cells and antigen presenting cells^55^. However, CCL20-CCR6 axis has also been shown to be crucial for cell trafficking during tissue repair and healing^56^.

This study is not without limitations. The use of intestinal cell culture models, including Caco-2 cell line and primary hSIEC, does not fully reproduce the cellular diversity and complex signalling dynamics of the intestinal mucosa *in vivo*. Therefore, validation of our findings in animal models or in more physiologically relevant systems, such as intestinal organoids, is necessary in future studies to confirm a translational relevance. Also, assays involving the primary hSIEC cells were mainly performed to validate our Caco-2 cell model and due to limited cell material, the number of replicate experiments were limited, which may affect the robustness of statistical comparisons. Furthermore, in this study, cells were exposed to bacterial components at a single post-chemotherapy time point (72 h). Extending the recovery period in the future experiments would allow evaluation of whether chemokine CXCL8 and CCL20 levels resolve. Lastly, the macrophage-related observations are correlative and do not prove a direct causal relationship between macrophage activation and barrier restoration. Future studies employing mechanistic approaches would help clarify their specific contribution.

Considering these limitations, it is essential to consider how our findings align with the available knowledge on probiotic-derived compounds in the context of chemotherapy. Current evidence regarding probiotics or their compounds usage concomitantly to cancer therapy is limited, particularly in patients undergoing chemotherapy treatment. As reviewed by others, many studies have reported beneficial effects of oral probiotic administration in treating various inflammatory disorders. However, the findings concerning probiotic benefits upon chemotherapy are constrained by the variability in preclinical studies and the lack of research involving immunocompromised animals^57^. Understanding the mechanisms behind the advantages of probiotics-derived compounds could significantly enhance cancer treatment management.

## Conclusions

Collectively, we demonstrate that probiotic bacteria-secreted components, such as the bioactive polysaccharide EPS synthesized by LR, have the potential to accelerate intestinal barrier recovery after chemotherapy exposure by reprogramming intracellular pathways critical for intestinal barrier maintenance and homeostasis. By enhancing barrier restoration while reshaping inflammatory and monocyte-related pathways, these microbial components highlight a promising way forward for mitigating gastrointestinal toxicity during chemotherapy.

## Supplementary Information

Below is the link to the electronic supplementary material.

Supplementary Material 1

## Data Availability

The datasets generated and/or analysed during the current study will be deposited in the National Center for Biotechnology Information (NCBI) Gene Expression Omnibus (GEO) upon acceptance of the manuscript and will be made publicly available immediately after publication. Other data is provided within the manuscript or supplemental information files.

## Abbreviations

5 FU: 5 Fluorouracil
BEA: Bioinformatics and expression analysis
CFS: Cell-free supernatant
CLDN1: Claudin-1
DEGs: Differentially expressed genes
DMEM: Dulbecco’s modified eagle medium
Doxo: Doxorubicin
EMEM: Essential medium eagle
EPS: Exopolysaccharides
ETEC: Enterotoxigenic *Escherichia coli*
FITC: Fluorescein isothiocyanate
GO: Gene ontology
hSIEC: human small intestinal epithelial cells
IBD: Inflammatory bowel disease
IEC: Intestinal epithelial cells
LGG: *Lacticaseibacillus rhamnosus* GG
LR: *Limosilactobacillus reuteri* DSM 17938
LTA: Lipoteichoic acid
MFI: Mean fluorescent intensity
MRS: De Man, Rogosa and Sharpe
MV: Membrane vesicles
NK: natural killer
OCLN: Occludin
PBMC: Peripheral blood mononuclear cells
Papp: Apparent permeability coefficient
PC: Principal components
PCA: Principal component analysis
PRR: Pattern recognition receptor
RA: Retinoic acid
TJP: Tight junction proteins
TEER: Transepithelial electrical resistance
ZO-1: Zonula occludens-1
