# Supplementary material for: Limosilactobacillus reuteri metabolites modulate immune pathways and intestinal barrier repair after 5 fluorouracil exposure
Source: Sci Rep. 2026 Apr 2;16:11376. doi: 10.1038/s41598-026-45524-y (PMC13049081; doi:10.1038/s41598-026-45524-y)
Supplement: Supplementary file 1 — Supplementary Material 1 [file 41598_2026_45524_MOESM1_ESM.docx]

**SUPPLEMENTARY DATA**


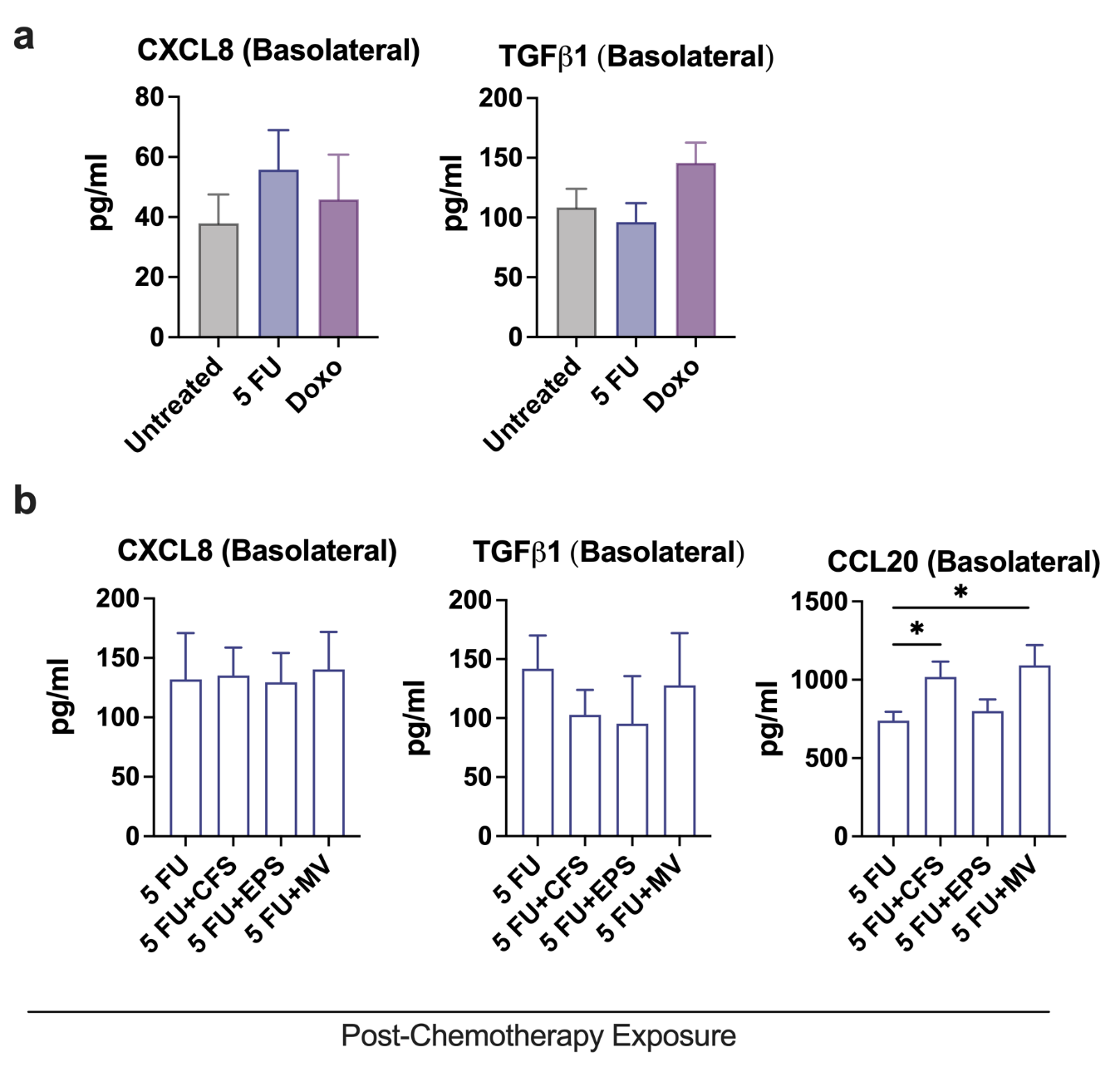


**Supplemental Figure 1. Basolateral protein secretion from Caco-2 cells with or without stimulation of bacterial components post-chemotherapy exposure.** Differentiated Caco-2 cells were exposed to 50 μg/ml of 5 FU or 1 μg/ml of Doxo for 24 h. Following chemotherapy removal, the cells were cultured with or without bacterial components for 72 h. **(a)** The graphs show soluble levels of CXCL8 and TGFβ1 in the supernatant of Caco-2 cells taken from the basolateral part of the culture insert after 5 FU or Doxo exposure. **(b)** Soluble levels of CXCL8, TGFβ1 and CCL20 after stimulation with bacterial components post-chemotherapy exposure. Results are presented as mean ± SEM from two independent experiments (n=4). Wilcoxon matched-pairs signed rank test was applied to determine statistical differences, *p <0.05.

**
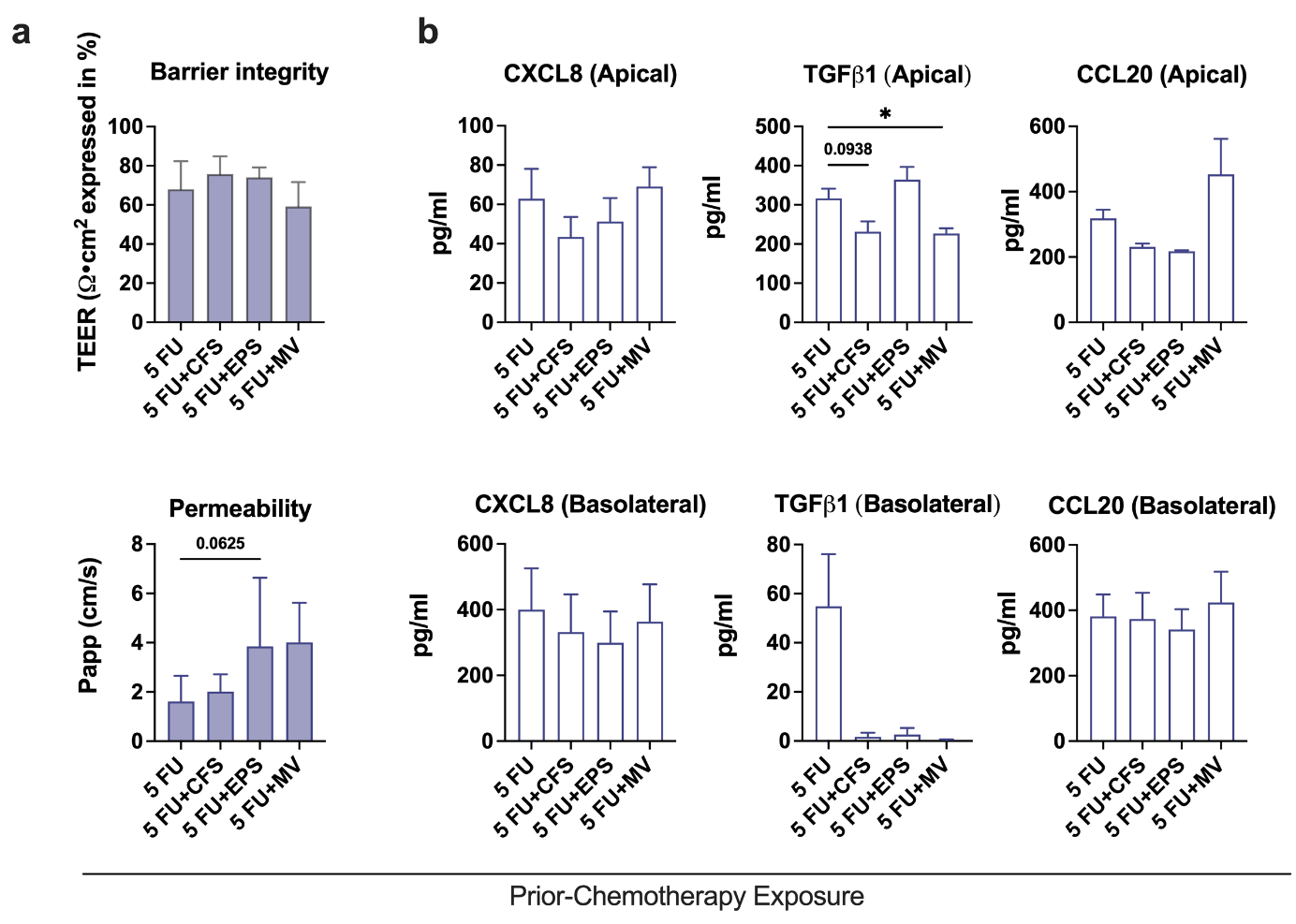
**

**Supplemental Figure 2. Caco-2 cell stimulation with bacterial components prior to 5 FU exposure.** Caco-2 cells were differentiated in the presence of bacterial components and then were exposed to 50 μg/ml of 5 FU for 24 h. Following chemotherapy removal, cells rested for 72 h. **(a)** The TEER values, measured as Ω.cm^2,^ and monolayer permeability, measured by FITC-dextran transport at cm/s, in Caco-2 cells. TEER values were corrected for the blank wells and then were expressed as percentage relative to the TEER values prior chemotherapy. **(b)** Apical and basolateral CXCL8, TGFβ1 and CCL20 secretion by Caco-2 cells. Results are presented as mean ± SEM from two independent experiments (n=3-6). Wilcoxon matched-pairs signed rank test was applied to determine statistical differences, *p <0.05.

**
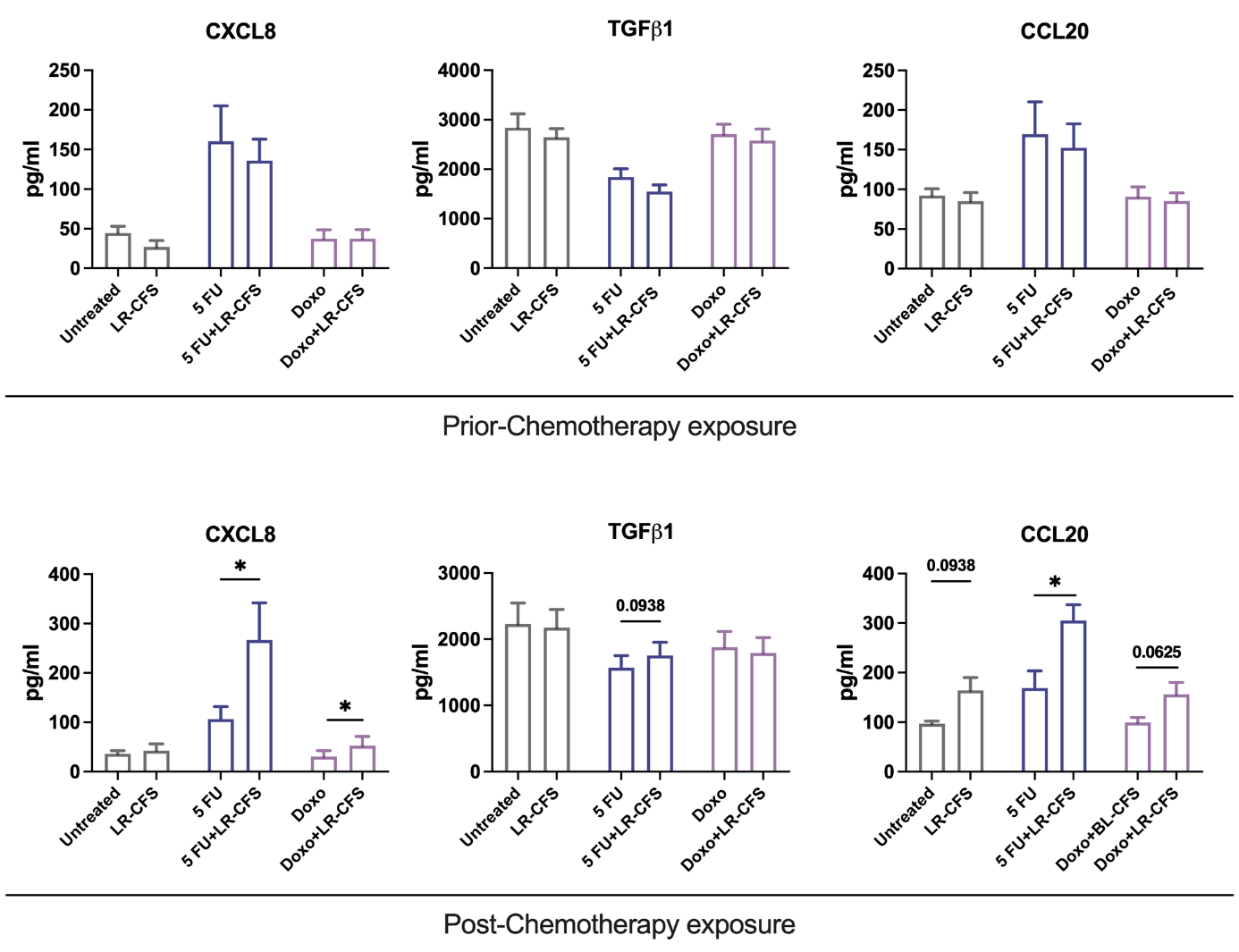
**

**Supplemental Figure 3. The effects of gut bacteria-derived cell-free supernatant on chemotherapy exposed undifferentiated Caco-2 cells.** Cells were either stimulated with LR-CFS and then were exposed to 5 FU or Doxo, or they were first exposed to chemotherapy drugs and then were stimulated with LR-CFS. The graphs show soluble CXCL8, TGFβ1 and CCL20 levels in the culture supernatant. Results are presented as mean ± SEM from two to three independent experiments (n=4-6). Wilcoxon matched-pairs signed rank test was applied to determine statistical differences, *p <0.05.


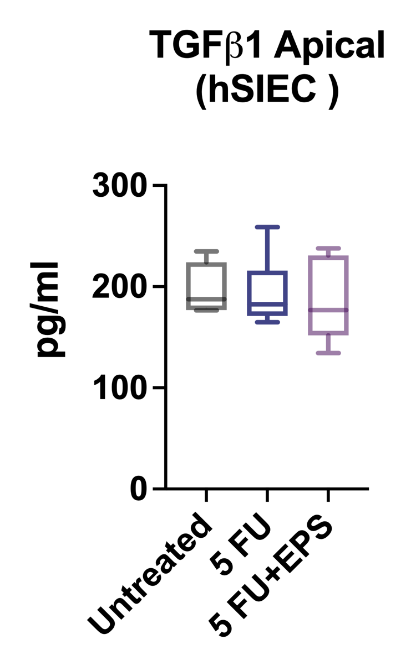


**Supplemental Figure 4. Apical TGFβ1 secretion** **from hSIEC** **upon stimulation with EPS post-chemotherapy exposure.** Differentiated hSIEC were exposed to 5 FU for 24 h. Following 5 FU removal, cells were cultured with EPS for 72 h and then the supernatant was collected for protein quantification. Results are presented as boxplots, displaying the range from minimum to maximum values (n=6).


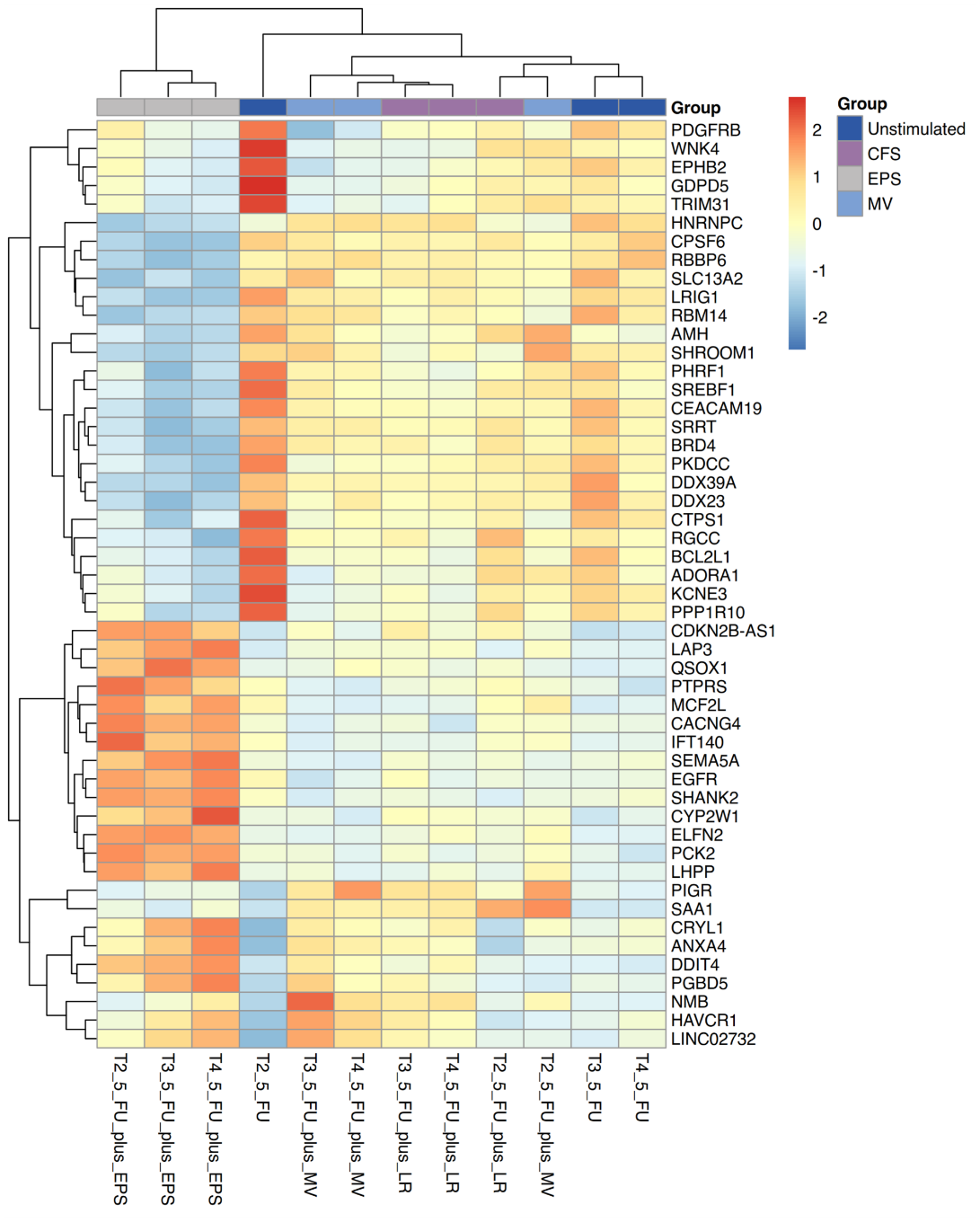


**Supplemental Figure 5. Heatmap of the Top 50 most significant differentially expressed genes.** Differentiated Caco-2 cells were exposed to 50 μg/ml of 5 FU for 24 h. Following 5 FU removal, cells were cultured with bacterial components (CFS, EPS and MV) for 72 h and then collected for RNA sequencing. A Heatmap showing top 50 differentially expressed genes, based on most significant in at least one of the three comparisons (CFS vs. Unstimulated, EPS vs. Unstimulated, or MV vs. Unstimulated). The expression values shown are variance stabilizing transformed (DESeq2) and scaled by row (Z-score).


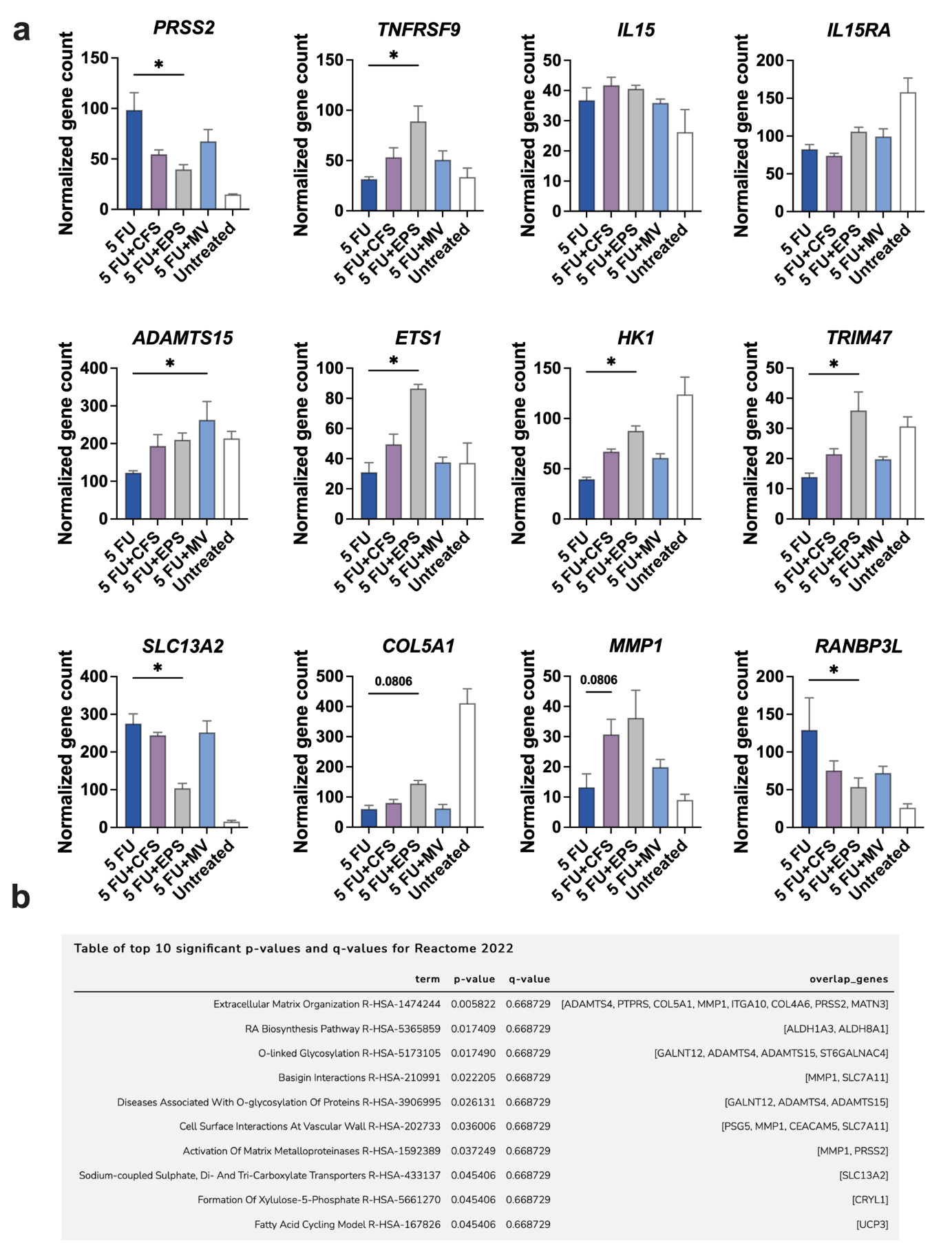


**Supplemental Figure 6. Transcriptomic changes in Caco-2 cells upon stimulation with bacterial components post-chemotherapy exposure.** Differentiated Caco-2 cells were exposed to 50 μg/ml of 5 FU for 24 h. Following 5 FU removal, cells were cultured with bacterial components for 72 h and then were collected for RNA-seq analysis. **(a)** Normalized gene counts for *PRSS2*, *TNFRSF9*, *IL15*, *IL15RA*, *ADAMTS15*, *ETS1*, *HK1*, *TRIM47*, *SLC13A2*, *COL5A1*, *MMP1* and *RANBP3L*, obtained from RNA-seq results. **(b)** Gene ontology analysis of biological processes associated with all significantly expressed genes in 5 FU exposed versus 5 FU exposed and EPS stimulated cells. The analysis was performed using an online tool Enrichr, while a companion application Appyter was employed for data visualization. Results are presented as mean ± SEM from two independent experiments (n=3). Paired Friedman test followed by Dunn´s multiple comparison was used to determine statistical difference. The 5 FU unexposed cells were not included in the statistical analysis to focus on the effects of bacterial components after 5 FU exposure, *p <0.05.


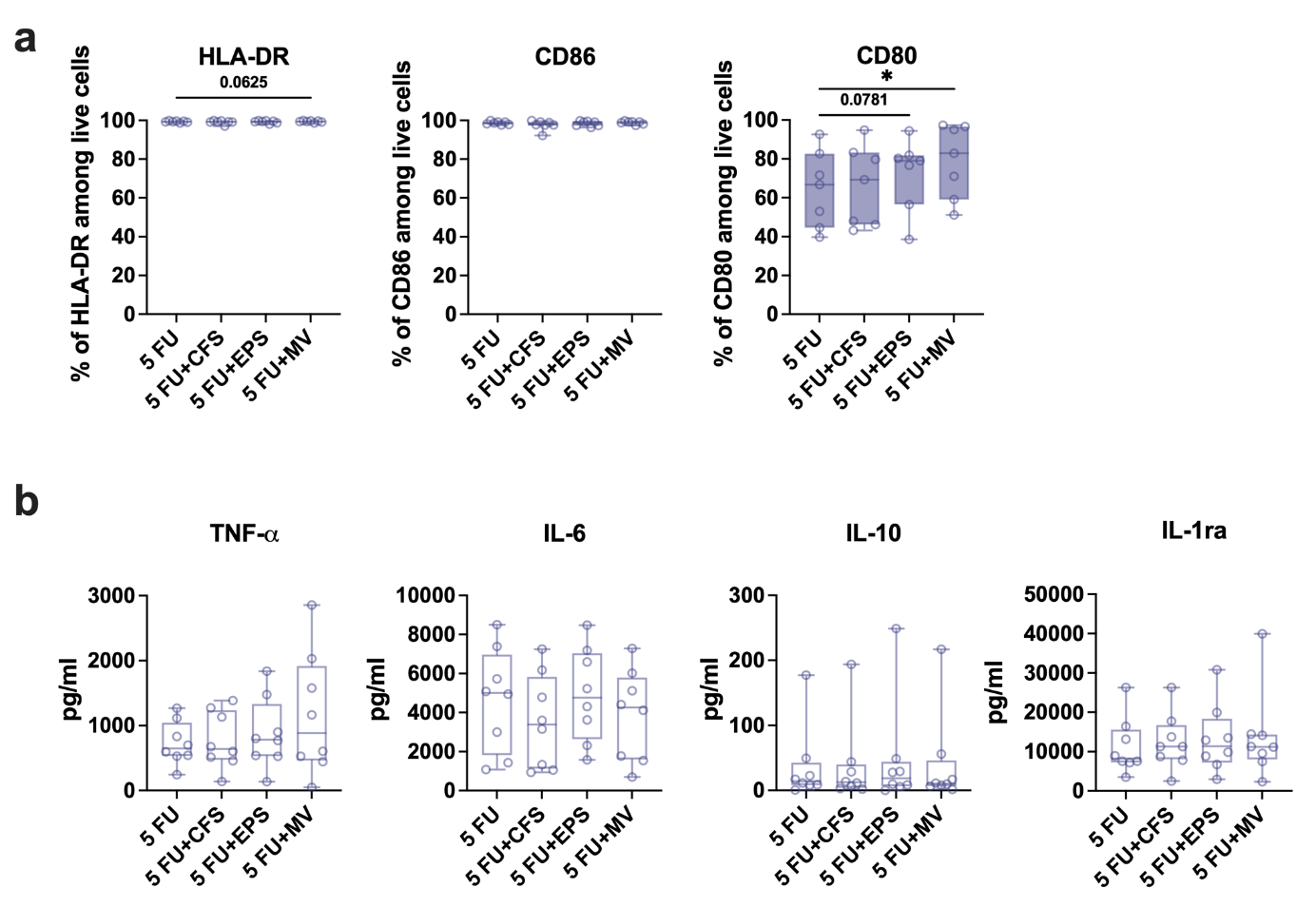


**Supplemental Figure 7. The phenotype and functional responses of macrophages polarized with Caco-2 cell supernatant.** Monocytes were cultured for 6 days in the presence of 5 FU exposed Caco-2 cell supernatant stimulated with either CFS, EPS or MV. On day 6, LPS was added for 24 h to activate macrophages. **(a)** The percentage of HLA-DR, CD86 and CD80 expressing macrophages on day 7 among all donors. **(b)** The secretion of TNF-α, IL-6, IL-10 and IL-1ra in the culture supernatant of polarized macrophages. Results are presented as boxplots, displaying the range from minimum to maximum values including all data points (n=7-8). Wilcoxon matched-pairs signed rank test was applied to determine statistical differences, *p <0.05.
